# MEASURING SPECIALTY CARE UTILIZATION AMONG MEDICALLY FRAGILE, LOW-INCOME OLDER OR DISABLED ADULTS

**DOI:** 10.1093/geroni/igac059.2841

**Published:** 2022-12-20

**Authors:** Kelly Goo, Mina Silberberg, Howard Eisenson, Fred Johnson, Sandra Stinnett

**Affiliations:** Duke University Medical Center, Durham, North Carolina, United States; Duke University Medical Center, Durham, North Carolina, United States; Duke University Medical Center, Durham, North Carolina, United States; Duke University Medical Center, Durham, North Carolina, United States; Duke University Medical Center, Durham, North Carolina, United States

## Abstract

The purpose of this research was to quantify the rates of specialty care referral completion and identify variables associated with successful completion among a population of low-income, elderly and/or disabled homebound patients with barriers to accessing office-based care. This was a cross-sectional study using descriptive and multivariate predictive analysis of specialty care referral completion, operationalized as attending an appointment of the same specialty care type within 6 months of being referred. Independent variables include patient age, sex, race, marital status, health insurance, blood pressure, and body mass index. Patient characteristics, referral information, and appointment information from July 1, 2014 to July 1, 2019 were extracted from electronic health record data of patients enrolled in the Just for Us primary care home visiting program in Durham, NC. Specialty care referrals were restricted to those for office-based consultations for chronic disease co-management originating from an outpatient primary care provider. Of 443 total referrals identified from 162 patients, 36% were successfully completed. Being married and female gender were found to be associated with successful referral completion. Of the 217 total patients in the study sample, 25% were identified as not having any referrals. No included patient characteristics were found to be significantly associated with being referred to specialty care. This study demonstrates that the specialty care needs of a medically and socially vulnerable population of homebound patients are not being adequately met.

